# Portrayal of mental health effects of isotretinoin on TikTok

**DOI:** 10.1016/j.jdin.2023.10.008

**Published:** 2023-12-01

**Authors:** Madison Hackley, Dalia Al-Bataineh, Jules B. Lipoff

**Affiliations:** Department of Dermatology, Lewis Katz School of Medicine, Temple University, Philadelphia, Pennsylvania

**Keywords:** Accutane, acne, depression, health misinformation, isotretinoin, mental health, social media, TikTok

*To the Editor:*

We read with interest the recent letter by Trepanowski et al,^1^ “Social media dermatologic advice: Dermatology without dermatologists.” The authors address how social media recommendations may not match recommendations by the American Academy of Dermatology. The public is increasingly turning to social media for dermatologic health information; eg, TikTok enables users to share video content, including about health conditions such as acne.^2^ Although this platform expands connectivity, it may also spread health misinformation.

Historically, isotretinoin has been associated with negative mental health side effects. The most reported such effects are depression and suicidal ideation.^3^ However, recent work suggests that psychiatric disturbances associated with isotretinoin may be less common than previously believed. A recent global population-based retrospective cohort study of >75,000 patients found those treated with isotretinoin had a lower risk of depression, bipolar disorder, and anxiety compared with those treated with systemic antibiotics.^4^ We aimed to characterize the representation of isotretinoin’s mental health effects on TikTok to examine if social media’s portrayal of psychiatric adverse events deviated from the most current literature.

We selected the first 50 TikTok videos that populated after searching for “Accutane” and “mental health” on TikTok’s platform. Excluding 4 videos that did not mention mental health, the >6000 video comments were initially imported from TikTok using Apify to scrape the data, which was then analyzed by reviewers (MH and DAB) for the comments’ overall sentiment toward isotretinoin’s risk of causing mental effects, classifying as describing high, medium, or low/no risk of mental side effects. Additionally, comments endorsing a high risk of negative mental effects were sorted by subcategory: depression, anxiety, suicide, or general mental health issues.

A majority of TikTok videos analyzed discussing isotretinoin’s mental health effects portrayed a high risk of negative effects (31/46, 67.4%). Similarly, 78.8% (249/316) of the comments on these videos suggested a high risk of negative mental health effects with isotretinoin. Notably, 10.8% (27/249) of high-risk comments mentioned suicide and 39.8% (99/249) mentioned depression. Additionally, 15.2% (7/46) of the videos were created by board-certified dermatologists, with the remaining 84.8% (39/46) of videos created by other users including other health-related professionals (pharmacists and estheticians). The kappa score for video reviewers was 0.64, indicating moderate agreement.

TikTok videos generally portray isotretinoin as having a high risk of psychiatric side effects, despite recent peer-reviewed evidence to the contrary. Depression is the second most common psychiatric concern associated with isotretinoin on TikTok, with general mental health concerns being the most common. Most videos discussing isotretinoin mental health effects were not made by board-certified dermatologists or other medical doctors, as has been seen in other social media.^1^ Our data were limited by sample size and moderate reviewer agreement. Still, the prevalence of potentially inaccurate information regarding isotretinoin’s mental health effects on TikTok may support greater involvement of health professionals or even artificial intelligence to scan for misinformation on social media.^5^

Future work may examine how to represent isotretinoin mental health risks more accurately in public forums, as misrepresentation could lead to unjustified concerns and prevent patients from seeking the medications they may need.

## Conflicts of interest

None disclosed.
